# Unraveling the causal association between inflammatory bowel diseases and uveitis through mendelian randomization analysis

**DOI:** 10.1038/s41598-025-90462-w

**Published:** 2025-02-16

**Authors:** Min Zhong, Hongjin An, Huatian Gan

**Affiliations:** 1 Department of Gastroenterology and Hepatology, West China Hospital, Sichuan University, Chengdu, China; 2 Department of Geriatrics and National Clinical Research Center for Geriatrics, West China Hospital, Sichuan University, Chengdu, China; 3 Department of Gastroenterology and Laboratory of Inflammatory Bowel Disease, The Center for Inflammatory Bowel Disease, Clinical Institute of Inflammation and Immunology, Frontiers Science Center for Disease-Related Molecular Network, West China Hospital, Sichuan University, Chengdu, China

**Keywords:** Inflammatory bowel disease, Crohn’s disease, Ulcerative colitis, Uveitis, Mendelian randomization, Causal relationship, Genetics, Diseases, Gastroenterology, Medical research, Risk factors

## Abstract

To investigate the causal relationship between inflammatory bowel disease (IBD) and uveitis, we conducted a two-sample bidirectional Mendelian randomization (MR) analysis utilizing summary data from genome-wide association studies (GWAS). The primary statistical analysis was performed using the inverse-variance weighted (IVW) method. False discovery rate (FDR) correction was used to control for false positives in multiple testing. In addition, sensitivity analyses were carried out using the MR Egger intercept test and Cochran’s Q test. The MR analysis revealed that genetically determined IBD (OR = 1.141, 95% CI 1.080–1.205, *P* = 2.21 × 10^−6^, P_FDR_ = 6.90 × 10^−6^), ulcerative colitis (UC) (OR = 1.113, 95% CI 1.032–1.201, *P* = 0.006, P_FDR_ = 0.009), and Crohn’s diseases (CD) (OR = 1.073, 95% CI 1.017–1.133, *P* = 0.010, P_FDR_ = 0.011) had a causal effect on uveitis. Conversely, the reverse MR analysis did not reveal significant causal link of uveitis on IBD, including its two subtypes. Furthermore, the results of the MR-Egger and weighted median methods were consistent with the IVW method. No evidence of heterogeneity or pleiotropy was detected by sensitivity analysis. Our findings confirm that IBD and its main subtypes had a causal connection with uveitis. Further research is needed to elucidate the underlying pathophysiological mechanisms driving this association.

**Supplementary Information:**

The online version contains supplementary material available at 10.1038/s41598-025-90462-w.

## Introduction

Inflammatory bowel disease (IBD), comprising Crohn’s disease (CD) and ulcerative colitis (UC), refers to a group of chronic immune-mediated intestinal illnesses^1^. Typical clinical symptoms in IBD patients include abdominal pain, diarrhea, chronic stomach pain, fatigue, and weight loss, as reported in numerous studies^2–4^. Besides gastrointestinal symptoms, IBD can manifest with extraintestinal manifestations (EIMs), primarily affecting the musculoskeletal, skin, and ocular system^5^. Studies have shown that EIMs occur in 6–47% of IBD patients, with around a quarter experiencing EIMs before IBD diagnosis^6–11^. The presence of EIMs significantly worsen the overall illness burden and negatively impact the quality of life of patients with IBD, sometimes surpassing the direct effects of the intestinal condition.

Uveitis, a group of inflammatory disorders affecting the uveal tract of the eye, which includes the iris, ciliary body, and choroid^12^. Even though ocular EIMs are not the most common IBD comorbidities, they can lead to serious consequences, such as vision loss. The pathogenic mechanisms of uveitis remain incompletely understood, leading to unclear preventive approaches and frequently disappointing treatment outcomes. This condition is often classified as either infected or non-infectious, with the non-infectious type being the most prevalent and primarily linked to autoimmune diseases. It frequently coexists with other autoimmune conditions, such as psoriasis, ankylosing spondylitis, and IBD^13–16^. A prospective study has suggested that uveitis subsequently occurred in 6.4% of CD patients and in 4.6% of UC patients during a 20-year follow-up period^17^. According to a different Swiss cohort study, the prevalence of uveitis was 5.6% in patients with UC and 11.1% in individuals with CD^18^. Furthermore, some studies have demonstrated that individuals with uveitis are substantially more likely than those without uveitis to develop IBD^19,20^. Despite the observation of a correlation between uveitis and IBD, causality has not been conclusively proven due to the possibility of reverse causality and the existence of unmeasured confounders that could undermine the relationship. From a clinical perspective, establishing causation is more critical for treatment and therapeutic decisions than establishing association.

Mendelian randomization (MR) is an epidemiological technique that uses genetic variants found in genome-wide association studies (GWAS) as instrumental variables (IVs) for exposure to evaluate the causal relationship between exposure and outcome^21^. Mendel’s second law, which asserts that parental alleles are randomly assigned to children during the meiosis process of gamete creation, independent of environmental, socioeconomic, or other confounding factors, is the foundation of the MR analysis. Therefore, this approach, which resembles a randomized controlled trial (RCT), are more likely to have causal interpretations^22^. MR analysis has been widely used in a variety of fields, including cardiovascular disease, pulmonary vascular diseases, mental health disorders, and others, enabling scientists to better understand the elements that contribute to the development of diseases^23–26^. Our research adopted a bidirectional MR analysis to ascertain the causal link between IBD, its subtypes (CD and UC), and uveitis.

## Methods

### Study design

**Fig. 1:**
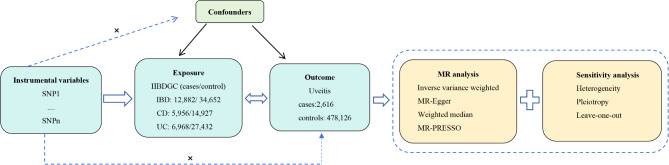
The schematic overview of the MR analysis. MR: Mendelian randomization; IBD: inflammatory bowel diseases; UC: Ulcerative colitis; CD: Crohn’s disease; PRESSO: Pleiotropy Residual Sum and Outlier.

To determine the correlation between IBD and uveitis, we conducted a bidirectional MR analysis following the principles of MR studies, which encompass three essential assumptions: (a) IVs display a significant correlation with exposure; (b) IVs are unaffected by confounding variables; (c) IVs solely affect the outcome through exposure (Fig. 1). The initial MR analysis explored the causal association between IBD and uveitis, while the subsequent MR analysis examined the reverse causality. The study was carried out according to the guidelines of the STROBE-MR^22^.

### Data sources

The summary data of IBD were obtained from the International Inflammatory Bowel Disease Genetics Consortium (IIBDGC)^27^, which was the largest genetic database for IBD to date, and included 12,882 IBD cases and 34,652 controls, 6,968 UC cases and 27,432 controls, and 5,956 CD cases and 14,927 controls. IBD and its subgroups were diagnosed using established endoscopic, histological, and radiological criteria. In addition, the genetic information relevant to uveitis was sourced from the IEU Open GWAS project (https://gwas.mrcieu.ac.uk), which encompassed 2,616 cases and 478,126 controls. This data was sourced from a meta-analysis of the UK Biobank (UKB) and FinnGen databases. The genetic background of these two study populations consisted mainly of individuals of European descent, and no overlapped populations between the exposures and the outcomes. No ethical permission was necessary for this study as our research data is derived from published research that has been authorized by the relevant ethical board.

### Instrumental variable selection

In this study, candidate single nucleotide polymorphisms (SNPs) strongly correlated with IBD, and uveitis were selected through several measures. Firstly, screening parameters for SNPs associated with IBD required a significance level of *P* < 5 × 10^−8^. Since none of the SNP associations with uveitis reached the genome-wide significance threshold (*P* < 5 × 10^−8^), the suggested significance level (*P* < 5 × 10^−6^) was used to extract the IVs, as has been adopted in previous MR analysis^28,29^. Secondly, we eliminated SNPs in linkage disequilibrium (LD) (r2 = 0.001, kb = 10 000) to guarantee the independence of the SNPs. Thirdly, the F statistic, calculated as [beta/SE]^2^, where beta means estimated effect size and se means standard error of beta, is commonly used to quantify bias in weak instrumental variables^30^. F-values less than 10 are not included in the analysis since they are regarded to be a sign of weak instrument bias^31^. Finally, selected SNPs linked to confounding factors, such as vitamin D, were deleted by scanning PhenoScannerV2 for potential associations^32^.

### Statistical analysis

Prior to analysis, we harmonized the summary data to ensure that each IV was aligned with the same effect alleles. Then, MR Steiger was used to examine the causality direction in SNPs, and those SNPs with the ‘TRUE’ direction were selected. The inverse variance weighted (IVW) was chosen as the main method for the MR analysis^31^. The weighted median and MR-Egger regression approaches were applied to assess the robustness of the outcomes^33,34^. In addition, the reliability of the results was evaluated using several sensitivity analyses. We calculate Cochran’s Q test, where *P* > 0.05 indicates no heterogeneity, and create an SNP funnel plots to display the MR results. Moreover, strong horizontal pleiotropy in the MR analysis is indicated if the MR-Egger intercept term in the analysis was statistically significant. To reduce the impact of outliers, MR pleiotropy residual sum and outlier (MR-PRESSO) eliminated any that were present and reanalyzed the data. Leave-one-out analysis was performed to find out if a single SNP was in charge of the entire effect. The statistical power of our MR investigation was then calculated using the online power calculator (mRnd) (https://github.com/kn3in/mRnd)^35^. Finally, false discovery rate (FDR) correction was used to control for false positives in multiple testing^36^.

Results were described by odds ratios (ORs) with 95% confidence intervals (CIs), and a P value of less than 0.05 was defined as significant. All data were performed using the “TwoSampleMR” (0.5.7) and “MRPRESSO” (1.0) packages in R software (Version 4.3.0).

## Results

### Selection of genetic variants

In the forward MR analysis, IBD had 62 independent genetic variants that were significant at the genome-wide level, whereas CD and UC had 51 and 37 genetic tools respectively (Supplementary Table 1). In addition, a total of 15 SNPs for the IBD group, 13 SNPs for UC, and 16 SNPs for CD were selected in the reverse MR analysis (Supplementary Table 2). Notably, each SNP included in the analyses exhibited an F-value exceeding 10, indicating a low risk of weak bias. The MR-PRESSO analyses revealed no outlier SNPs.

### Causal effect of IBD and its main subtypes on uveitis

**Fig. 2:**
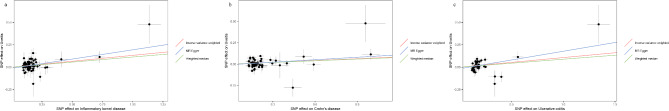
Scatter plots of MR analysis showing the effect of IBD and its two subtypes on uveitis. (**a**) Analysis of IBD on uveitis; (**b**) Analysis of CD on uveitis; (**c**) Analysis of UC on uveitis. The x-axes represent the genetic instrument-IBD associations, and the y-axes represent genetic instrument-anxiety associations. Black dots denote the genetic instruments included in the MR analysis. Red: Inverse-variance weighted; Green: Weighted median; blue: MR Egger. IBD: inflammatory bowel diseases; CD: Crohn’s disease; UC: Ulcerative colitis; SNP: Single-nucleotide polymorphism.

Genetically predicted that IBD (OR = 1.141, 95% CI: 1.080–1.205, *P* = 2.21 × 10^−6^, P_FDR_ = 6.90 × 10^−6^) had a strong causal relationship with the risk of uveitis through the IVW method (Table 1; Fig. 2). Additionally, the weighted median (OR = 1.120, 95% CI: 1.024–1.225, *P* = 0.013, P_FDR_ = 0.027) and MR Egger (OR = 1.038, 95% CI: 1.038–1.456, *P* = 0.020, P_FDR_ = 0.050) approaches yielded similar conclusions. Table 1 also provides more details of the results of the sensitivity analyses. In details, there was no heterogeneity between the SNPs as the p-value of Cochran’s Q statistics was above 0.05. Furthermore, the MR-Egger regression’s intercept was utilized to assess the reliability of our horizontal pleiotropy. The results indicated no horizontal pleiotropy in the causative impact (*P* > 0.05, Table 1). The funnel plot also shows no signs of symmetry departure (Supplementary Fig. 1). Additionally, the leave-one-out strategy did not reveal any specific SNP exerting a major influence on the outcomes (Supplementary Fig. 2). More importantly, IBD had sufficient statistical power (power > 0.8) for uveitis (Supplementary Fig. 3).

**Table 1:** The result of MR analysis between IBD and uveitis.

Exposure	Outcome	SNPs (*n*)	IVW			MR Egger			Weighted median			Cochran Q test *P* value	MR-Egger Intercept *P* value	Global test *P* value
			OR (95% CI)	*P* value	*P*_FDR_	OR (95% CI)	*P* value	*P*_FDR_	OR (95% CI)	*P* value	*P*_FDR_			
IBD	uveitis	62	1.141 (1.080–1.205)	2.21E-06	6.90E-06	1.229 (1.038–1.456)	0.020	0.050	1.120 (1.024–1.225)	0.013	0.027	0.083	0.358	0.079
CD	uveitis	51	1.073 (1.017–1.133)	0.010	0.011	1.101 (0.965–1.256)	0.158	0.123	1.068 (0.996–1.145)	0.065	0.044	0.028	0.678	0.025
UC	uveitis	37	1.113 (1.032–1.201)	0.006	0.009	1.215 (0.967–1.527)	0.103	0.111	1.093 (1.000-1.195)	0.050	0.042	0.006	0.431	0.009
Uveitis	IBD	15	1.011 (0.963–1.061)	0.662	0.418	1.019 (0.950–1.094)	0.603	0.304	1.022 (0.957–1.092)	0.510	0.208	0.888	0.753	0.832
Uveitis	CD	16	0.994 (0.932–1.060)	0.846	0.478	0.974 (0.885–1.071)	0.594	0.301	0.956 (0.879–1.039)	0.288	0.140	0.985	0.583	0.976
Uveitis	UC	13	1.033 (0.971-1.100)	0.303	0.247	1.062 (0.973–1.160)	0.206	0.130	1.041 (0.948–1.143)	0.395	0.169	0.696	0.403	0.487

MR, Mendelian randomization analysis; SNPs, Number of single nucleotide polymorphism; IVW, inverse-variance weighted; IBD, inflammatory bowel disease; CD, Crohn’s disease; UC, ulcerative colitis; FDR, false discovery rate; OR, Odds Ratio; CI, Confidence Interval.

The investigation into the two primary subtypes of IBD, UC and CD, uncovered a causal link with uveitis, with UC (OR = 1.113, 95% CI: 1.032–1.201, *P* = 0.006, P_FDR_ = 0.009) and CD (OR = 1.073, 95% CI: 1.017–1.133, *P* = 0.010, P_FDR_ = 0.011) (Table 1; Fig. 2). The weighted median and MR-Egger analyses supported these findings (Table 1). In the sensitivity analyses, no pleiotropy was found by the intercepts of the MR‒Egger regression (*P* > 0.05). The MR-PRESSO global test showed a statistical significance (CD: *p* = 0.025, UC: *p* = 0.009) for the connection with uveitis, but additional investigations did not reveal any significant horizontal pleiotropic outliers. In addition, Cochran’s Q statistic was employed to detect heterogeneity. As shown in Table 1, the Cochran’s Q statistic revealed heterogeneity among the IVs (CD: *p* = 0.028, UC: *p* = 0.006), which could be due to data from different consortium, but it has no bearing on the main conclusions of the study. Supplementary Fig. 1 showed the funnel plots of SNPs linked with CD and UC on the risk of uveitis. The leave-one-out analysis confirmed that none of the individual IVs singularly accounted for outcomes (Supplementary Fig. 2). The results of power analysis were shown in Supplementary Fig. 3, and all statistical power rates were greater than 80%.

### Causal effect of uveitis on IBD and its main subtypes

**Fig. 3:**
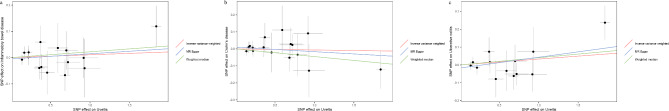
Scatter plots of MR analysis showing the effect of uveitis on IBD and its two subtypes. (**a**) Analysis of uveitis on IBD; (**b**) Analysis of uveitis on CD; (**c**) Analysis of uveitis on UC. The x-axes represent the genetic instrument-IBD associations, and the y-axes represent genetic instrument-anxiety associations. Black dots denote the genetic instruments included in the MR analysis. Red: Inverse-variance weighted; Green: Weighted median; blue: MR Egger. IBD: inflammatory bowel diseases; CD: Crohn’s disease; UC: Ulcerative colitis; SNP: Single-nucleotide polymorphism.

In the reverse stage, we investigated the causal connection between uveitis as exposure data and the risk of IBD and its subtypes. Utilizing the IVW approach, our results revealed no causal association between uveitis and IBD (OR = 1.011, 95% CI: 0.963–1.061, *P* = 0.662, P_FDR_ = 0.418), UC (OR = 1.033, 95% CI: 0.971-1.100, *P* = 0.303, P_FDR_ = 0.247), and CD (OR = 0.994, 95% CI: 0.932–1.060, *P* = 0.846, P_FDR_ = 0.478) (Table 1; Fig. 3). These findings were further supported by additional complementary methods (Table 1), demonstrating the absence of a causal link. The MR-Egger intercept test was performed to increase the robustness of these findings, and the results revealed no horizontal pleiotropy (*P* > 0.05, Table 1). Additionally, heterogeneity was measured by Cochran’s Q statistic, and Table 1 revealed that there was no significant statistical heterogeneity in the effect of uveitis on IBD. Scatter plots illustrating this analysis can be found in Supplementary Fig. 4. Moreover, the leave-one-out analysis indicated that no single SNP significantly altered the observed causal relationship between uveitis and IBD and its subtypes (Supplementary Fig. 5). However, uveitis did not have sufficient statistical power for IBD, CD as well as UC in the reverse analysis (power < 0.8) (Supplementary Fig. 3).

## Discussion

In our study, we conducted the first investigation into the potential causative link between IBD and uveitis utilizing the bidirectional MR analysis. Our findings demonstrated that IBD and its two subtypes had an increased risk of uveitis. However, reverse MR study showed that there is no causative relationship of uveitis to IBD and its two subtypes.

Uveitis is a common ocular manifestation in patients with IBD, with previous epidemiological studies suggesting a potential connection between the two conditions. Within the IBD population, uveitis occurs in 5–10% of cases, with a higher prevalence observed in patients with CD compared to those with UC^37,38^. For example, the prevalence of uveitis was more significant in patients with CD than in patients with UC (7.4% in CD versus 1.7% in UC) in a retrospective analysis involving 595 patients with UC and 216 with CD^39^. Furthermore, Vavricka et al. revealed an association between uveitis and CD activity but not with UC^8^. Additionally, a meta-analysis also confirmed that IBD patients, particularly those with CD, have a higher likelihood of developing uveitis, with CD patients exhibiting approximately 1.6 times the risk of uveitis compared to UC patients^40^. Our MR study further suggested that individuals with genetic susceptibility to both UC and CD are at increased risk of uveitis.

Limited studies have been conducted on the order in which uveitis and IBD occur. Aletaha et al. investigated a large commercial health insurance claims database and found a significantly higher risk of an IBD event among those with uveitis compared to the non-uveitis group^20^. Another retrospective cohort study involving 198,923 patients with uveitis also revealed a significantly higher cumulative incidence of IBD in the uveitis group when compared to controls^19^. Observational studies face challenges in establishing causal relationships due to confounding factors that influence exposure and outcome. Utilizing genetic instrumental variables in MR analysis can help mitigate the impact of these confounding factors and generate more precise causal estimates. In our study, we found no causal link between uveitis and IBD when uveitis was used as an exposure variable.

The mechanism underling the correlation between IBD and uveitis remains unclear. Das et al. suggested that shared epitopes between the intestinal mucosa and the uvea may play a role in the pathogenesis of both diseases^41^. Additionally, the onset and course of the diseases between IBD and uveitis are influenced by genetic factors to a certain extent. One study showed that extraintestinal manifestations in 70% of parent-offspring pairs and 84% of sibling pairs, highlighting the role of genotype^42^. The first identified risk variant in CD patients, NOD2/CARD15, has also been associated with uveitis, suggesting a common pathogenic mechanism underlying uveitis and IBD development^43^. Furthermore, the connections between the gut and the eyes may be significantly influenced by the gut flora, as demonstrated by the positive effect of oral short-chain fatty acid therapy on experimental autoimmune uveitis severity^44^. However, further research is required to ascertain whether genetic factors, alterations in the gut microbiome, or shifts in immune responses are the primary drivers of the connections between IBD and uveitis.

This study has several advantages. Firstly, this is the first study that we are aware of that uses GWAS data to investigate the causal connection between IBD and uveitis, making it less prone to reverse causation and confounding factors than observational research. Secondly, in order to avoid demographic stratification, our study population was limited to individuals of European heritage. Thirdly, we conducted sensitivity analyses to ensure the dependability of the outcomes and consistency in causal estimates. However, our study also entails several limitations. We emphasize that our analytical power was relatively limited in observing the effect of uveitis on IBD, which might be due in part to the lower proportion of uveitis variability explained by valid SNPs. Furthermore, the MR analysis solely included participants of European descent and the results may not be applicable to other groups. IBD incidence varies by ancestry and geography, with lower rates in Asia and greater rates in North America and Europe^45^. Also, the occurrence of uveitis may be ancestry-related^46,47.^ Additionally, the substantial differences in population characteristics between the two samples, such as age, gender, and socioeconomic background, could impact the validity of causal inference.

## Conclusions

Our study conclusively suggested that IBD and its two subtypes have a positive causal effect on uveitis, but not vice versa. Understanding the ocular manifestations in IBD patients is essential from a clinical perspective. Timely recognition of ocular EIMs in patients with IBD allows for successful treatment without subsequent complications. By establishing a causal relationship between IBD and uveitis, this study lays a crucial groundwork for future research aimed at identifying common mechanisms and potential therapeutic targets.

## Electronic supplementary material

Below is the link to the electronic supplementary material.

Supplementary Material 1

## Data Availability

GWAS summary statistics are available from IEU (https://gwas.mrcieu.ac.uk/) database.
