# Supplementary material for: Unraveling the causal association between inflammatory bowel diseases and uveitis through mendelian randomization analysis
Source: Sci Rep. 2025 Feb 16;15:5686. doi: 10.1038/s41598-025-90462-w (PMC11830775; doi:10.1038/s41598-025-90462-w)
Supplement: Supplementary file 1 — Supplementary Material 1 [file 41598_2025_90462_MOESM1_ESM.docx]

**Supplementary Figures**

**
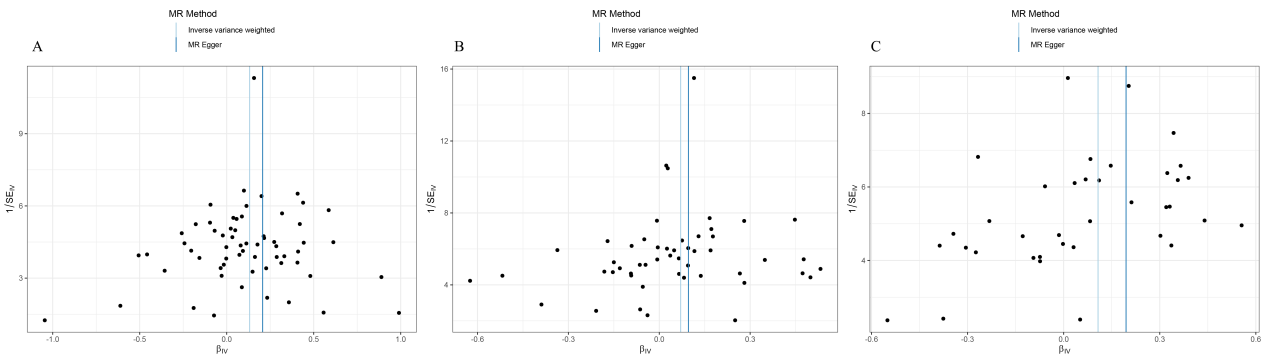
**

**Supplementary Fig. 1**. Funnel plot of SNPs associated with IBD and its two subtypes on the risk of uveitis. (A) Analysis of IBD on uveitis; (B) Analysis of CD on uveitis; (C) Analysis of UC on uveitis. IBD, inflammatory bowel disease; CD, Crohn's disease; UC, ulcerative colitis.


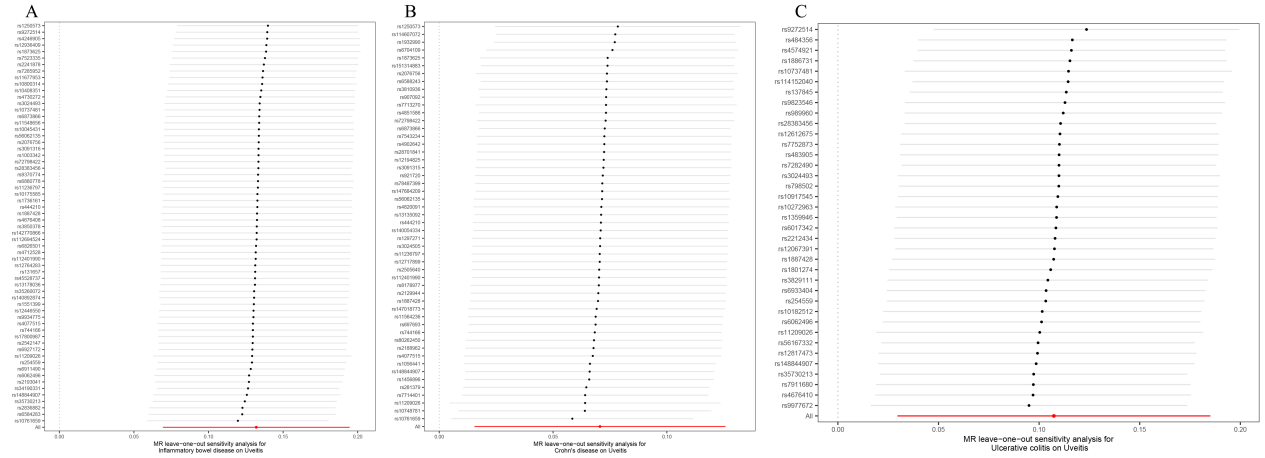


**Supplementary Fig. 2**. Leave-one-out analysis of IBD and its two subtypes on uveitis. (A) Analysis of IBD on uveitis; (B) Analysis of CD on uveitis; (C) Analysis of UC on uveitis. IBD, inflammatory bowel disease; CD, Crohn's disease; UC, ulcerative colitis.

**
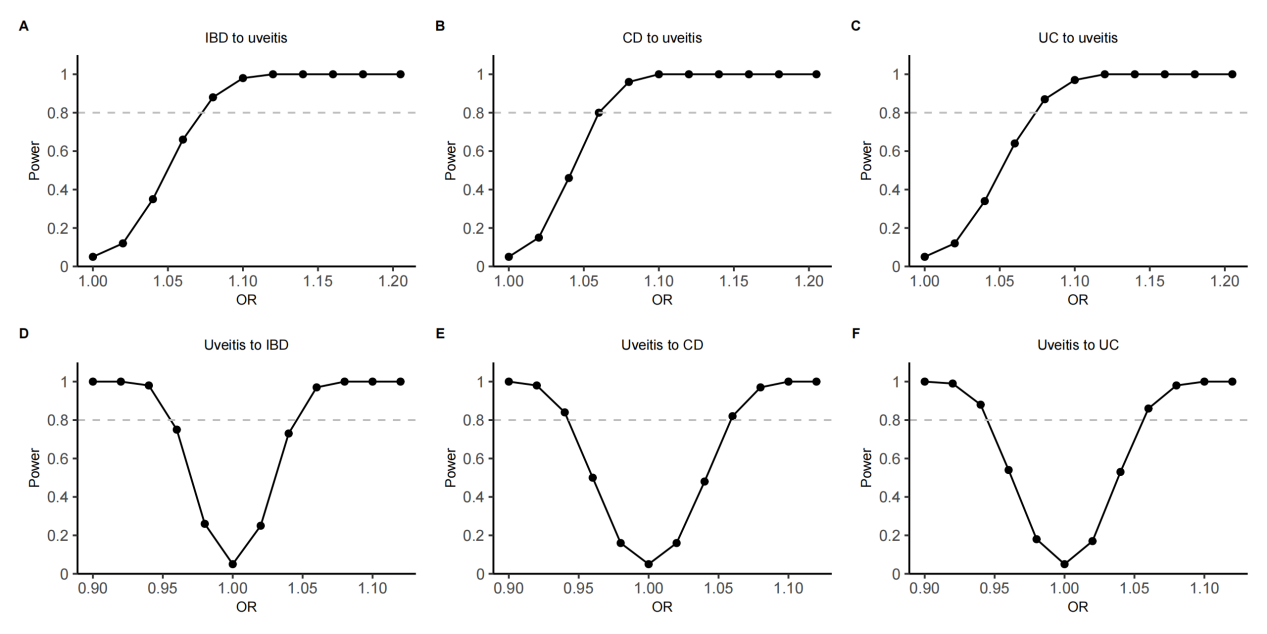
Supplementary Fig. 3**. The statistical power for Mendelian randomization analysis. The dashed grey line indicates a statistical power of 0.8. (A) IBD to uveitis; (B) CD to uveitis; (C) UC to uveitis; (D) uveitis to IBD; (E) uveitis to CDs; (F) uveitis to UC. IBD: Inflammatory Bowel Disease; CD: Crohn’s Disease; UC: Ulcerative Colitis; OR: odds ratio.


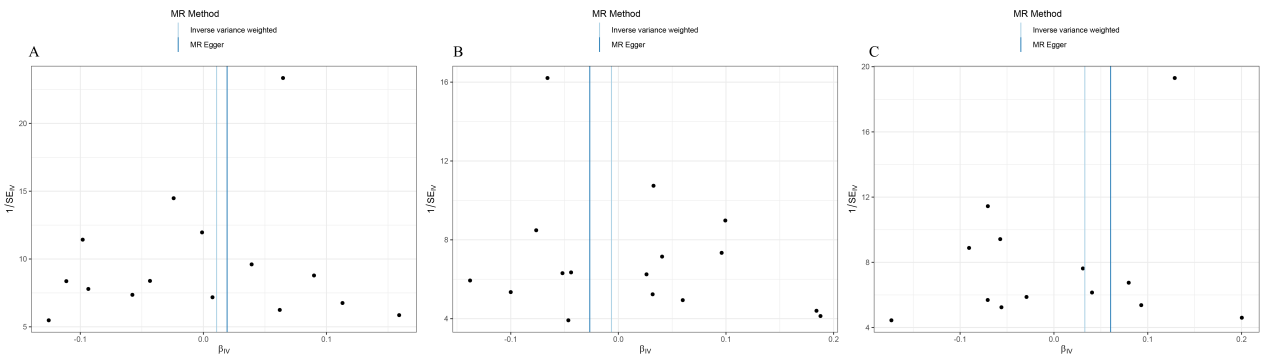


**Supplementary Fig. 4**. Funnel plot of SNPs associated with uveitis on the risk of IBD, CD, and UC. (A) Analysis of uveitis on IBD; (B) Analysis of uveitis on CD; (C) Analysis of uveitis on UC. IBD, inflammatory bowel disease; CD, Crohn's disease; UC, ulcerative colitis.


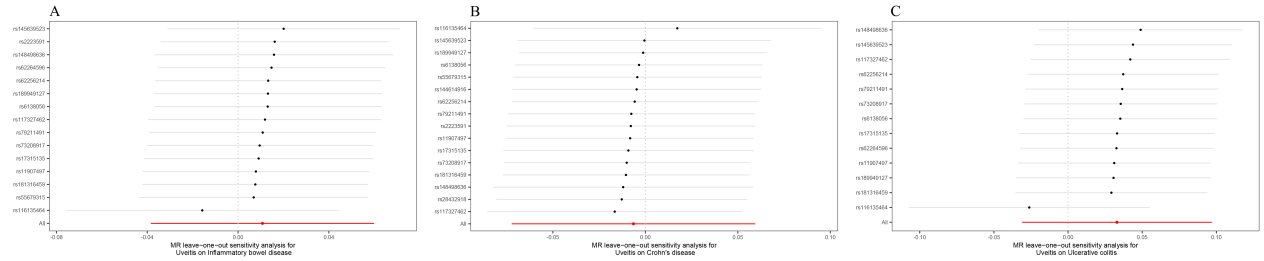


**Supplementary Fig. 5**. Leave-one-out analysis of uveitis on the risk of IBD, CD, and UC. (A) Analysis of uveitis on IBD; (B) Analysis of uveitis on CD; (C) Analysis of uveitis on UC. IBD, inflammatory bowel disease; CD, Crohn's disease; UC, ulcerative colitis.

**Supplementary Tables**

**Supplementary Table 1**. Characteristics of genetic variants used to estimate the effect of IBD and its two subtypes on uveitis.

| **SNP** | **effect allele** | **other allele** | **F** | **Exposure** | | | **Outcome** | | |
| --- | --- | --- | --- | --- | --- | --- | --- | --- | --- |
|  |  |  |  | **beta** | **se** | **p** | **beta** | **se** | **p** |
| IBD | | | | | | | | | |
| rs1003342 | G | A | 31.977 | -0.095 | 0.017 | 1.67E-08 | 0.003 | 0.028 | 0.905 |
| rs10045431 | C | A | 88.108 | 0.177 | 0.019 | 6.59E-21 | 0.010 | 0.033 | 0.755 |
| rs10175585 | A | G | 43.482 | -0.133 | 0.020 | 3.89E-11 | 0.025 | 0.076 | 0.738 |
| rs10408351 | A | G | 38.881 | 0.138 | 0.022 | 4.23E-10 | -0.022 | 0.036 | 0.547 |
| rs10737481 | G | T | 68.893 | 0.141 | 0.017 | 1.19E-16 | 0.003 | 0.028 | 0.906 |
| rs10761659 | G | A | 88.596 | 0.162 | 0.017 | 4.07E-21 | 0.095 | 0.028 | 0.001 |
| rs10800314 | A | C | 63.908 | -0.143 | 0.018 | 1.17E-15 | 0.010 | 0.029 | 0.732 |
| rs11209026 | A | G | 296.207 | -0.726 | 0.042 | 1.76E-66 | -0.114 | 0.064 | 0.076 |
| rs11236797 | A | C | 83.888 | 0.156 | 0.017 | 4.75E-20 | 0.014 | 0.028 | 0.627 |
| rs112401990 | A | G | 66.790 | 0.142 | 0.017 | 2.84E-16 | 0.033 | 0.065 | 0.611 |
| rs112694524 | A | G | 38.622 | 0.188 | 0.030 | 5.39E-10 | 0.016 | 0.072 | 0.819 |
| rs11548656 | G | A | 33.351 | -0.293 | 0.051 | 7.72E-09 | 0.001 | 0.068 | 0.993 |
| rs11677953 | A | G | 32.576 | 0.098 | 0.017 | 1.05E-08 | -0.035 | 0.030 | 0.238 |
| rs12446550 | A | G | 39.739 | 0.108 | 0.017 | 2.78E-10 | 0.031 | 0.028 | 0.264 |
| rs1250573 | A | G | 35.752 | -0.114 | 0.019 | 2.21E-09 | 0.058 | 0.029 | 0.046 |
| rs12764283 | A | G | 50.020 | 0.127 | 0.018 | 1.57E-12 | 0.022 | 0.029 | 0.438 |
| rs12936409 | T | C | 75.215 | 0.146 | 0.017 | 3.87E-18 | -0.026 | 0.028 | 0.352 |
| rs131657 | A | T | 41.454 | 0.136 | 0.021 | 1.24E-10 | 0.049 | 0.068 | 0.475 |
| rs13178036 | C | G | 30.073 | 0.101 | 0.018 | 4.13E-08 | 0.023 | 0.030 | 0.440 |
| rs140892874 | C | T | 63.998 | 0.410 | 0.051 | 1.28E-15 | 0.088 | 0.088 | 0.315 |
| rs142770866 | A | G | 46.580 | 0.230 | 0.034 | 8.14E-12 | 0.026 | 0.052 | 0.616 |
| rs148844907 | A | T | 139.524 | 1.138 | 0.096 | 3.63E-32 | 0.478 | 0.217 | 0.027 |
| rs1551399 | C | A | 34.288 | 0.101 | 0.017 | 5.01E-09 | 0.100 | 0.065 | 0.123 |
| rs1736161 | A | G | 50.213 | -0.123 | 0.017 | 1.34E-12 | -0.010 | 0.028 | 0.725 |
| rs17800987 | G | A | 43.733 | 0.202 | 0.031 | 3.71E-11 | 0.055 | 0.045 | 0.219 |
| rs1873625 | A | C | 98.111 | 0.177 | 0.018 | 3.71E-23 | -0.017 | 0.029 | 0.572 |
| rs1887428 | C | G | 92.933 | -0.172 | 0.018 | 6.65E-22 | -0.020 | 0.029 | 0.496 |
| rs2076756 | G | A | 101.722 | 0.188 | 0.019 | 5.59E-24 | 0.009 | 0.038 | 0.807 |
| rs2193041 | G | A | 60.426 | 0.134 | 0.017 | 6.91E-15 | 0.059 | 0.030 | 0.048 |
| rs2241878 | C | T | 76.693 | 0.148 | 0.017 | 1.75E-18 | -0.014 | 0.028 | 0.611 |
| rs2542147 | T | G | 44.425 | -0.151 | 0.023 | 2.78E-11 | -0.050 | 0.039 | 0.195 |
| rs254559 | A | C | 35.721 | 0.103 | 0.017 | 2.08E-09 | 0.042 | 0.028 | 0.139 |
| rs2836882 | A | G | 95.373 | -0.196 | 0.020 | 1.49E-22 | -0.086 | 0.032 | 0.007 |
| rs28383456 | T | C | 78.767 | -0.178 | 0.020 | 5.77E-19 | 0.186 | 0.142 | 0.192 |
| rs3024493 | A | C | 92.053 | 0.213 | 0.022 | 8.48E-22 | 0.008 | 0.039 | 0.835 |
| rs3091316 | A | G | 34.691 | -0.112 | 0.019 | 3.59E-09 | 0.000 | 0.030 | 0.991 |
| rs34190331 | A | G | 34.085 | 0.177 | 0.030 | 5.39E-09 | 0.157 | 0.058 | 0.007 |
| rs35260072 | C | A | 69.965 | 0.142 | 0.017 | 7.07E-17 | 0.030 | 0.030 | 0.315 |
| rs35730213 | C | G | 60.908 | -0.151 | 0.019 | 6.91E-15 | -0.093 | 0.034 | 0.006 |
| rs3850378 | C | T | 30.250 | 0.155 | 0.028 | 3.80E-08 | -0.011 | 0.107 | 0.916 |
| rs4077515 | T | C | 108.791 | 0.179 | 0.017 | 1.50E-25 | 0.036 | 0.028 | 0.200 |
| rs4246905 | C | T | 68.462 | 0.163 | 0.020 | 1.42E-16 | -0.042 | 0.034 | 0.208 |
| rs444210 | G | A | 42.484 | 0.110 | 0.017 | 7.39E-11 | 0.008 | 0.028 | 0.772 |
| rs45528737 | T | C | 30.914 | 0.167 | 0.030 | 2.66E-08 | 0.093 | 0.106 | 0.382 |
| rs4676408 | A | G | 42.576 | 0.118 | 0.018 | 6.62E-11 | 0.011 | 0.029 | 0.701 |
| rs4712528 | C | G | 35.077 | 0.123 | 0.021 | 3.07E-09 | 0.020 | 0.032 | 0.528 |
| rs4730272 | G | A | 56.758 | -0.134 | 0.018 | 4.50E-14 | 0.003 | 0.028 | 0.915 |
| rs56062135 | T | C | 58.083 | 0.151 | 0.020 | 2.64E-14 | 0.005 | 0.032 | 0.880 |
| rs6062496 | A | G | 84.025 | 0.165 | 0.018 | 5.48E-20 | 0.053 | 0.029 | 0.070 |
| rs6584283 | C | T | 113.824 | -0.180 | 0.017 | 1.70E-26 | -0.074 | 0.028 | 0.008 |
| rs6826501 | T | C | 30.149 | -0.093 | 0.017 | 4.12E-08 | -0.014 | 0.028 | 0.627 |
| rs6873866 | C | T | 36.958 | -0.107 | 0.018 | 1.09E-09 | 0.065 | 0.058 | 0.258 |
| rs6880778 | G | A | 117.843 | 0.188 | 0.017 | 2.14E-27 | 0.019 | 0.028 | 0.511 |
| rs6911490 | C | T | 47.134 | -0.143 | 0.021 | 6.82E-12 | -0.059 | 0.035 | 0.092 |
| rs6927172 | G | C | 29.813 | 0.110 | 0.020 | 4.65E-08 | 0.053 | 0.036 | 0.138 |
| rs72798422 | C | T | 41.484 | 0.278 | 0.043 | 1.19E-10 | -0.005 | 0.078 | 0.953 |
| rs7285952 | G | T | 56.092 | -0.176 | 0.024 | 7.60E-14 | 0.036 | 0.043 | 0.398 |
| rs744166 | G | A | 49.247 | -0.121 | 0.017 | 2.16E-12 | -0.034 | 0.028 | 0.218 |
| rs7523335 | A | G | 38.995 | -0.141 | 0.023 | 4.16E-10 | 0.034 | 0.032 | 0.280 |
| rs9272514 | T | C | 127.319 | -0.235 | 0.021 | 1.59E-29 | 0.107 | 0.059 | 0.068 |
| rs9370774 | C | T | 35.620 | -0.131 | 0.022 | 2.54E-09 | 0.004 | 0.042 | 0.928 |
| rs9934775 | T | C | 36.205 | -0.140 | 0.023 | 1.71E-09 | -0.044 | 0.039 | 0.256 |
| CD | | | | | | | | | |
| rs1056441 | C | T | 42.890 | 0.167 | 0.026 | 5.44E-11 | 0.058 | 0.031 | 0.060 |
| rs10748781 | A | C | 84.750 | -0.219 | 0.024 | 3.72E-20 | -0.061 | 0.029 | 0.034 |
| rs10761659 | G | A | 80.020 | 0.212 | 0.024 | 3.42E-19 | 0.095 | 0.028 | 0.001 |
| rs11209026 | A | G | 242.559 | -0.995 | 0.064 | 1.05E-54 | -0.114 | 0.064 | 0.076 |
| rs11236797 | A | C | 61.466 | 0.181 | 0.023 | 4.85E-15 | 0.014 | 0.028 | 0.627 |
| rs112401990 | A | G | 31.116 | 0.132 | 0.024 | 2.35E-08 | 0.033 | 0.065 | 0.611 |
| rs114607072 | T | G | 49.334 | 0.442 | 0.063 | 2.20E-12 | -0.276 | 0.105 | 0.008 |
| rs11564236 | T | A | 76.113 | 0.519 | 0.060 | 2.85E-18 | 0.088 | 0.088 | 0.317 |
| rs12194825 | A | T | 33.277 | -0.172 | 0.030 | 8.00E-09 | 0.008 | 0.034 | 0.817 |
| rs1250573 | A | G | 41.904 | -0.171 | 0.026 | 9.01E-11 | 0.058 | 0.029 | 0.046 |
| rs12717899 | T | G | 30.344 | 0.159 | 0.029 | 3.59E-08 | 0.013 | 0.036 | 0.720 |
| rs1297271 | T | C | 42.718 | -0.155 | 0.024 | 6.28E-11 | -0.010 | 0.028 | 0.724 |
| rs13135092 | G | A | 32.421 | 0.221 | 0.039 | 1.21E-08 | -0.014 | 0.084 | 0.867 |
| rs140054334 | T | C | 31.025 | 0.350 | 0.063 | 2.57E-08 | -0.014 | 0.151 | 0.928 |
| rs1456896 | T | C | 30.801 | 0.139 | 0.025 | 2.90E-08 | 0.066 | 0.030 | 0.028 |
| rs147018773 | T | C | 73.593 | 0.322 | 0.038 | 8.89E-18 | 0.042 | 0.048 | 0.385 |
| rs147684209 | C | T | 40.302 | 0.155 | 0.024 | 2.34E-10 | -0.032 | 0.061 | 0.595 |
| rs148844907 | A | T | 45.579 | 0.958 | 0.142 | 1.47E-11 | 0.478 | 0.217 | 0.027 |
| rs151314883 | A | G | 46.922 | -0.224 | 0.033 | 7.12E-12 | 0.034 | 0.043 | 0.430 |
| rs1873625 | A | C | 55.300 | 0.181 | 0.024 | 1.09E-13 | -0.017 | 0.029 | 0.572 |
| rs1887428 | C | G | 47.854 | -0.168 | 0.024 | 4.22E-12 | -0.020 | 0.029 | 0.496 |
| rs1932990 | T | C | 33.799 | 0.153 | 0.026 | 6.02E-09 | -0.079 | 0.034 | 0.020 |
| rs2076756 | G | A | 272.940 | 0.400 | 0.024 | 3.24E-61 | 0.009 | 0.038 | 0.807 |
| rs2129944 | G | T | 33.222 | -0.156 | 0.027 | 7.81E-09 | -0.021 | 0.035 | 0.538 |
| rs2188962 | T | C | 86.782 | 0.212 | 0.023 | 1.36E-20 | 0.037 | 0.030 | 0.223 |
| rs2505640 | G | A | 37.794 | -0.146 | 0.024 | 7.61E-10 | -0.014 | 0.029 | 0.631 |
| rs281379 | A | G | 34.502 | 0.140 | 0.024 | 4.26E-09 | 0.074 | 0.029 | 0.009 |
| rs28701841 | A | G | 36.162 | 0.224 | 0.037 | 1.85E-09 | -0.021 | 0.050 | 0.675 |
| rs3024505 | A | G | 34.702 | 0.178 | 0.030 | 3.90E-09 | 0.012 | 0.039 | 0.764 |
| rs3091315 | G | A | 46.582 | -0.180 | 0.026 | 9.52E-12 | 0.001 | 0.030 | 0.976 |
| rs3810936 | C | T | 62.427 | 0.208 | 0.026 | 2.46E-15 | -0.010 | 0.032 | 0.746 |
| rs4077515 | T | C | 84.406 | 0.216 | 0.024 | 4.37E-20 | 0.036 | 0.028 | 0.200 |
| rs444210 | G | A | 50.915 | 0.163 | 0.023 | 1.02E-12 | 0.008 | 0.028 | 0.772 |
| rs4820091 | G | T | 37.073 | 0.172 | 0.028 | 1.22E-09 | 0.006 | 0.031 | 0.839 |
| rs4851586 | C | T | 41.877 | -0.169 | 0.026 | 9.94E-11 | 0.022 | 0.034 | 0.520 |
| rs4902642 | A | G | 29.970 | -0.129 | 0.024 | 4.34E-08 | 0.012 | 0.028 | 0.665 |
| rs56062135 | T | C | 51.528 | 0.193 | 0.027 | 7.45E-13 | 0.005 | 0.032 | 0.880 |
| rs6588243 | C | A | 31.679 | 0.132 | 0.023 | 1.78E-08 | -0.024 | 0.028 | 0.390 |
| rs6704109 | T | C | 62.263 | 0.202 | 0.026 | 2.77E-15 | -0.035 | 0.031 | 0.272 |
| rs6873866 | C | T | 49.467 | -0.168 | 0.024 | 2.06E-12 | 0.065 | 0.058 | 0.258 |
| rs697693 | A | G | 37.596 | 0.172 | 0.028 | 8.36E-10 | 0.048 | 0.042 | 0.247 |
| rs72798422 | C | T | 135.068 | 0.590 | 0.051 | 3.19E-31 | -0.005 | 0.078 | 0.953 |
| rs744166 | G | A | 30.795 | -0.129 | 0.023 | 2.92E-08 | -0.034 | 0.028 | 0.218 |
| rs7543234 | T | C | 33.918 | 0.155 | 0.027 | 6.10E-09 | -0.010 | 0.030 | 0.741 |
| rs7713270 | T | C | 151.466 | 0.297 | 0.024 | 6.97E-35 | 0.008 | 0.028 | 0.769 |
| rs7714401 | A | T | 42.679 | 0.159 | 0.024 | 6.20E-11 | 0.076 | 0.029 | 0.010 |
| rs78487399 | C | G | 37.276 | 0.226 | 0.037 | 1.03E-09 | -0.012 | 0.058 | 0.832 |
| rs80262450 | A | G | 64.319 | 0.283 | 0.035 | 1.08E-15 | 0.050 | 0.042 | 0.236 |
| rs8178977 | C | G | 49.512 | 0.193 | 0.027 | 2.06E-12 | 0.018 | 0.032 | 0.565 |
| rs907092 | A | G | 32.708 | 0.130 | 0.023 | 1.01E-08 | -0.020 | 0.028 | 0.471 |
| rs921720 | G | A | 47.241 | 0.163 | 0.024 | 6.40E-12 | -0.001 | 0.030 | 0.971 |
| UC | | | | | | | | | |
| rs10182512 | A | G | 51.995 | 0.161 | 0.022 | 5.19E-13 | 0.052 | 0.030 | 0.081 |
| rs10272963 | T | C | 63.338 | -0.172 | 0.022 | 1.69E-15 | -0.012 | 0.028 | 0.670 |
| rs10737481 | G | T | 134.071 | 0.250 | 0.022 | 4.37E-31 | 0.003 | 0.028 | 0.906 |
| rs10917545 | A | G | 30.530 | -0.185 | 0.034 | 3.29E-08 | 0.000 | 0.042 | 0.993 |
| rs11209026 | A | G | 118.039 | -0.562 | 0.052 | 1.58E-27 | -0.114 | 0.064 | 0.076 |
| rs114152040 | A | G | 29.714 | 0.340 | 0.062 | 4.95E-08 | -0.104 | 0.078 | 0.184 |
| rs12067391 | T | G | 32.872 | 0.163 | 0.029 | 9.67E-09 | 0.008 | 0.068 | 0.902 |
| rs12612675 | G | A | 31.494 | 0.123 | 0.022 | 1.98E-08 | -0.012 | 0.030 | 0.701 |
| rs12817473 | G | A | 77.230 | 0.191 | 0.022 | 1.71E-18 | 0.062 | 0.030 | 0.039 |
| rs1359946 | A | G | 34.631 | 0.158 | 0.027 | 3.84E-09 | 0.005 | 0.036 | 0.893 |
| rs137845 | G | A | 31.085 | 0.118 | 0.021 | 2.38E-08 | -0.032 | 0.028 | 0.247 |
| rs148844907 | A | T | 151.704 | 1.341 | 0.109 | 7.17E-35 | 0.478 | 0.217 | 0.027 |
| rs1801274 | G | A | 71.038 | -0.183 | 0.022 | 3.78E-17 | -0.027 | 0.028 | 0.334 |
| rs1886731 | C | T | 40.418 | -0.141 | 0.022 | 2.25E-10 | 0.033 | 0.028 | 0.241 |
| rs1887428 | C | G | 62.227 | -0.177 | 0.022 | 3.36E-15 | -0.020 | 0.029 | 0.496 |
| rs2212434 | T | C | 44.381 | 0.142 | 0.021 | 2.46E-11 | 0.012 | 0.028 | 0.677 |
| rs254559 | A | C | 33.427 | 0.124 | 0.022 | 7.63E-09 | 0.042 | 0.028 | 0.139 |
| rs28383456 | T | C | 173.608 | -0.337 | 0.026 | 1.07E-39 | 0.186 | 0.142 | 0.192 |
| rs3024493 | A | C | 73.299 | 0.236 | 0.028 | 1.09E-17 | 0.008 | 0.039 | 0.835 |
| rs35730213 | C | G | 46.462 | -0.167 | 0.025 | 8.81E-12 | -0.093 | 0.034 | 0.006 |
| rs3829111 | A | G | 53.347 | 0.156 | 0.021 | 2.89E-13 | 0.033 | 0.028 | 0.237 |
| rs4574921 | T | C | 34.607 | 0.151 | 0.026 | 4.24E-09 | -0.058 | 0.034 | 0.088 |
| rs4676410 | A | G | 53.538 | 0.208 | 0.028 | 2.46E-13 | 0.076 | 0.032 | 0.016 |
| rs483905 | A | G | 31.964 | 0.129 | 0.023 | 1.57E-08 | -0.010 | 0.032 | 0.770 |
| rs484356 | G | C | 34.644 | -0.134 | 0.023 | 3.95E-09 | 0.046 | 0.028 | 0.104 |
| rs56167332 | A | C | 43.068 | 0.152 | 0.023 | 5.30E-11 | 0.067 | 0.030 | 0.025 |
| rs6017342 | C | A | 63.538 | 0.191 | 0.024 | 1.38E-15 | 0.016 | 0.028 | 0.573 |
| rs6062496 | A | G | 50.067 | 0.158 | 0.022 | 1.47E-12 | 0.053 | 0.029 | 0.070 |
| rs6933404 | C | T | 43.811 | 0.167 | 0.025 | 3.68E-11 | 0.050 | 0.036 | 0.159 |
| rs7282490 | A | G | 42.615 | -0.140 | 0.021 | 7.08E-11 | 0.053 | 0.058 | 0.364 |
| rs7752873 | T | C | 36.197 | 0.182 | 0.030 | 1.83E-09 | -0.014 | 0.045 | 0.762 |
| rs7911680 | C | A | 65.056 | -0.172 | 0.021 | 8.27E-16 | -0.067 | 0.028 | 0.015 |
| rs798502 | C | A | 32.617 | -0.136 | 0.024 | 1.21E-08 | 0.002 | 0.029 | 0.944 |
| rs9272514 | T | C | 226.235 | -0.402 | 0.027 | 4.00E-51 | 0.107 | 0.059 | 0.068 |
| rs9823546 | A | T | 62.928 | 0.177 | 0.022 | 2.29E-15 | -0.010 | 0.029 | 0.727 |
| rs989960 | T | C | 36.054 | -0.129 | 0.022 | 1.77E-09 | 0.017 | 0.028 | 0.552 |
| rs9977672 | A | G | 88.120 | -0.245 | 0.026 | 6.21E-21 | -0.084 | 0.033 | 0.010 |

SNP, single-nucleotide polymorphisms; SE, standard error of SNP effect; IBD, inflammatory bowel diseases; CD, Crohn’s diseases; UC, ulcerative colitis.

**Supplementary Table 2**. Characteristics of genetic variants used to estimate the effect of uveitis on IBD and its two subtypes.

| **SNP** | **effect allele** | **other allele** | **F** | **Exposure** | | | **Outcome** | | |
| --- | --- | --- | --- | --- | --- | --- | --- | --- | --- |
|  |  |  |  | **beta** | **se** | **p** | **beta** | **se** | **p** |
| IBD | | | | | | | | | |
| rs116135464 | T | C | 869.210 | 1.840 | 0.062 | 7.55E-191 | 0.119 | 0.079 | 0.131 |
| rs117327462 | A | C | 24.528 | 0.919 | 0.186 | 7.31E-07 | -0.001 | 0.077 | 0.990 |
| rs11907497 | C | T | 24.553 | -0.166 | 0.033 | 7.24E-07 | -0.019 | 0.025 | 0.445 |
| rs145639523 | T | C | 92.845 | 0.682 | 0.071 | 5.52E-22 | -0.067 | 0.060 | 0.262 |
| rs148498636 | G | A | 22.396 | 0.720 | 0.152 | 2.22E-06 | -0.017 | 0.050 | 0.725 |
| rs17315135 | T | C | 21.932 | 0.699 | 0.149 | 2.81E-06 | 0.027 | 0.073 | 0.707 |
| rs181316459 | C | G | 28.994 | 0.371 | 0.069 | 7.34E-08 | 0.059 | 0.063 | 0.352 |
| rs189949127 | T | G | 21.066 | 0.931 | 0.203 | 4.44E-06 | -0.040 | 0.111 | 0.716 |
| rs2223591 | A | G | 26.637 | 0.356 | 0.069 | 2.47E-07 | -0.040 | 0.043 | 0.351 |
| rs55679315 | G | A | 21.634 | 0.221 | 0.048 | 3.38E-06 | 0.020 | 0.025 | 0.428 |
| rs6138056 | G | A | 21.536 | 0.137 | 0.030 | 3.55E-06 | -0.008 | 0.019 | 0.671 |
| rs62256214 | A | C | 22.422 | 0.456 | 0.096 | 2.20E-06 | -0.057 | 0.083 | 0.491 |
| rs62264596 | C | A | 21.417 | 0.382 | 0.083 | 3.72E-06 | -0.036 | 0.049 | 0.467 |
| rs73208917 | T | C | 21.288 | 0.592 | 0.128 | 3.94E-06 | 0.037 | 0.095 | 0.698 |
| rs79211491 | C | A | 29.085 | 0.217 | 0.040 | 6.93E-08 | 0.002 | 0.030 | 0.958 |
| CD | | | | | | | | | |
| rs116135464 | T | C | 869.210 | 1.840 | 0.062 | 7.55E-191 | -0.121 | 0.114 | 0.286 |
| rs117327462 | A | C | 24.528 | 0.919 | 0.186 | 7.31E-07 | 0.091 | 0.102 | 0.372 |
| rs11907497 | C | T | 24.553 | -0.166 | 0.033 | 7.24E-07 | -0.010 | 0.034 | 0.767 |
| rs144614916 | C | A | 39.777 | 0.787 | 0.125 | 2.85E-10 | -0.035 | 0.124 | 0.780 |
| rs145639523 | T | C | 92.845 | 0.682 | 0.071 | 5.52E-22 | -0.052 | 0.080 | 0.517 |
| rs148498636 | G | A | 22.396 | 0.720 | 0.152 | 2.22E-06 | 0.024 | 0.067 | 0.726 |
| rs17315135 | T | C | 21.932 | 0.699 | 0.149 | 2.81E-06 | 0.028 | 0.098 | 0.772 |
| rs181316459 | C | G | 28.994 | 0.371 | 0.069 | 7.34E-08 | 0.068 | 0.084 | 0.418 |
| rs189949127 | T | G | 21.066 | 0.931 | 0.203 | 4.44E-06 | -0.128 | 0.157 | 0.414 |
| rs2223591 | A | G | 26.637 | 0.356 | 0.069 | 2.47E-07 | 0.009 | 0.057 | 0.870 |
| rs28432918 | A | G | 34.875 | -0.181 | 0.031 | 3.55E-09 | -0.017 | 0.025 | 0.480 |
| rs55679315 | G | A | 21.634 | 0.221 | 0.048 | 3.38E-06 | -0.012 | 0.035 | 0.743 |
| rs6138056 | G | A | 21.536 | 0.137 | 0.030 | 3.55E-06 | -0.014 | 0.026 | 0.593 |
| rs62256214 | A | C | 22.422 | 0.456 | 0.096 | 2.20E-06 | -0.021 | 0.116 | 0.855 |
| rs73208917 | T | C | 21.288 | 0.592 | 0.128 | 3.94E-06 | 0.111 | 0.143 | 0.438 |
| rs79211491 | C | A | 29.085 | 0.217 | 0.040 | 6.93E-08 | 0.007 | 0.041 | 0.867 |
| UC | | | | | | | | | |
| rs116135464 | T | C | 869.210 | 1.840 | 0.062 | 7.55E-191 | 0.237 | 0.095 | 0.013 |
| rs117327462 | A | C | 24.528 | 0.919 | 0.186 | 7.31E-07 | -0.053 | 0.098 | 0.590 |
| rs11907497 | C | T | 24.553 | -0.166 | 0.033 | 7.24E-07 | -0.015 | 0.031 | 0.617 |
| rs145639523 | T | C | 92.845 | 0.682 | 0.071 | 5.52E-22 | -0.062 | 0.077 | 0.422 |
| rs148498636 | G | A | 22.396 | 0.720 | 0.152 | 2.22E-06 | -0.051 | 0.063 | 0.421 |
| rs17315135 | T | C | 21.932 | 0.699 | 0.149 | 2.81E-06 | 0.022 | 0.092 | 0.814 |
| rs181316459 | C | G | 28.994 | 0.371 | 0.069 | 7.34E-08 | 0.074 | 0.081 | 0.357 |
| rs189949127 | T | G | 21.066 | 0.931 | 0.203 | 4.44E-06 | 0.074 | 0.138 | 0.591 |
| rs6138056 | G | A | 21.536 | 0.137 | 0.030 | 3.55E-06 | -0.004 | 0.023 | 0.865 |
| rs62256214 | A | C | 22.422 | 0.456 | 0.096 | 2.20E-06 | -0.079 | 0.103 | 0.441 |
| rs62264596 | C | A | 21.417 | 0.382 | 0.083 | 3.72E-06 | 0.015 | 0.062 | 0.803 |
| rs73208917 | T | C | 21.288 | 0.592 | 0.128 | 3.94E-06 | -0.033 | 0.113 | 0.769 |
| rs79211491 | C | A | 29.085 | 0.217 | 0.040 | 6.93E-08 | -0.015 | 0.038 | 0.688 |

SNP, single-nucleotide polymorphisms; SE, standard error of SNP effect; IBD, inflammatory bowel diseases; CD, Crohn’s diseases; UC, ulcerative colitis.
